# Improving Financial Literacy Using the Medical Mini-MBA at a Canadian Medical School

**DOI:** 10.7759/cureus.25595

**Published:** 2022-06-02

**Authors:** Eric Poon, Paul Bissonnette, Sina Sedighi, Wyatt MacNevin, Ketan Kulkarni

**Affiliations:** 1 Faculty of Medicine, Dalhousie University, Halifax, CAN; 2 Medicine, Dalhousie University, Halifax, CAN; 3 Hematology and Oncology, Queen Elizabeth II Health Sciences Centre, Halifax, CAN

**Keywords:** medical student, financial planning, wellness, postgraduate education, financial literacy, undergraduate medical education

## Abstract

Introduction

Financial literacy correlates with less debt and better retirement planning. Medical students, residents, and physicians often have poor financial literacy and large amounts of debt. We measured baseline financial literacy and whether it improved with the administration of a financial literacy course.

Methods

We created the Medical Mini-MBA,a six-week financial literacy course that targeted gaps in financial literacy among medical students and residents. Weekly topics included personal finance, investing, real estate and mortgage, physician billing and payment models, income and tax, and choosing a medical specialty. A 46-question financial literacy assessment was delivered to participants before and after the course.

Results

Of the 276 who participated in the course, 179 (64.86%) participated in the study. Participants who completed the course improved their financial literacy score by 10.10/46.00±5.12 (n=93, p<0.001). Self-assessment of financial literacy was positively correlated with financial literacy exam scores (r=0.366, p<0.001). Demographics such as gender, geography, education level, and first-degree relatives who are/were physicians had no effect on financial literacy scores.

Conclusions

The Medical Mini-MBA improved financial literacy at a Canadian medical school. Implementation of the coursemay equip medical students and residents for financial decisions. It avoids financial conflicts of interest and can supplement the medical curriculum.

## Introduction

Canadian medical students often have large amounts of debt. Twenty-nine point eight percent (29.80%) of students have debt ranging from $80,000-$140,000 and 13.10% have debt greater than $200,000 [1]. Several years of residency training after graduating medical school with high debt is stressful and is associated with worse academic performance [2]. Financial literacy is defined as the knowledge, skills, and confidence to make responsible financial decisions [3]. Medical students have been found to have poor financial literacy throughout medical education despite school-provided personal financial counseling or information [3-4]. On the contrary, good financial literacy has been shown to be associated with medical students incurring less debt and being more likely to invest for retirement [4]. These reports of poor financial literacy have also been documented in residents, which suggests that financial education is often not prioritized in medical education [5-8]. Furthermore, current approaches to personal financial counseling by medical schools are ineffective and not associated with improved financial literacy in medical students, as poor self-perception of financial literacy and skills continues in practice [4,9-10]

Implementation of a finance-focused mini-course alongside the medical curriculum may improve financial literacy. Previous studies show a need in the areas of budgeting, debt management, income and billing efficiency, estate planning, investment strategies, and retirement planning [6,8,11-12]. Although financial literacy interventions are needed for medical students and residents in Canada, there currently does not exist a comprehensive financial literacy course to address this issue.

To address the need for improved financial literacy for Canadian medical students and residents, we developed the Medical Mini-MBA (MMM) implemented at Dalhousie Medical School. This study aims to address the limited data on financial literacy in medical trainees while also investigating the impact of the MMM on improving the financial literacy of medical students and residents.

## Materials and methods

We performed a single-arm interventional study. Research ethics board approval was attained by Dalhousie Health Sciences Research Ethics Board (File No. 2020-5397). The MMM was created as an online course delivered both synchronously and asynchronously for six consecutive weeks. Instructors taught with video and displayed content material. The six-week course ran for one hour each week and covered six different weekly topics: personal finance, investing, physician payment models, real estate and mortgage, income and tax, and specialty salaries and career choice (Table 1). A pre-intervention and post-intervention survey, with the financial literacy exam included, were distributed to study participants using Opinio (Object Plant, Oslo, Norway).

**Table 1:** Medical Mini-MBA course syllabus RRSP: registered retirement savings plan; TFSA: tax-free savings account; ETF: exchange-traded fund; REIT: real estate investment trust

Week & Date	Topics and learning objectives
Week 1	Financial literacy & personal finance in medical school residency. Financial myths reducing debt incomes & investing overview. Social determinants of wealth
Week 2	Investing Introduction to capital markets: what are stocks and bonds? How markets work (efficient market hypothesis). Why is investing important? A sensible approach to investing. Active vs. passive empirical evidence. Practical implications of efficient market theory. Concentration vs. diversification. Asset allocation: stocks, bonds, cash, debt, and human capital. Asset location: RRSP, TFSA, taxable, corporation. Housing decision: rent vs. buy and the 5% rule. Where to find financial advice DIY online research. Pitfalls of individual investors. Fee-based financial advice. Robo-advisors. Asset allocation ETFs
Week 3	Real estate & mortgage. Buy vs rent. Price-rent ratio. Loan, mortgage, and insurance. What can I afford? Real estate market REITs. Passive income
Week 4	How physicians are paid: introduction to payment models. Academic vs community practice remuneration models pros & cons. How much do I earn in each model? MD “side hustle”: non-clinical work as medical students, residents, and staff
Week 5	Income & tax. Early stage of career. Gain an understanding of personal tax & credits available. Middle stage of career. How to minimize tax burden during years earning high income. Late stage of career. How to plan for retirement to preserve wealth
Week 6	Choosing your specialty: remuneration and employment factors. Income lifestyle. Employment landscape and projection

Participants were coded with study identification numbers to enable paired comparisons post-intervention. Demographic data were acquired in the pre-intervention survey. Consent forms to participate were procured and participant data were de-identified for privacy and confidentiality.

Population

All medical students and resident physicians at the Dalhousie Faculty of Medicine were invited to enroll in the course and study in Autumn 2020. Course enrollment information was distributed using medical school social media groups and official medical education correspondence for registration. Upon registering for the course, participants were invited to participate in the concurrent study. Participation was voluntary and had no impact on the ability to attend and complete the MMM.

Course development

For instructor recruitment, we sought Canadian financial experts and educators. Financial conflicts of interest were disclosed by the instructors and no financial products were advertised. Instructors taught voluntarily and were unpaid, with no direct nor indirect remuneration opportunities available. Lecturers were not provided with any contact information for the participants. The instructional content was reviewed prior to the delivery of the course. Each lecture was 50 minutes of instruction followed by 10 minutes of participant questions.

Study participants were asked to complete a pre-intervention survey before the start of the MMM and a post-intervention survey two weeks after course completion. Participants were given two months to complete the post-intervention survey to accommodate medical school schedules.

Survey structure

Instructors created 46 multiple-choice questions for the financial literacy examination. Their questions were respective to their instructional topics in the course, which aligned with course learning objectives and content. These questions were reviewed for consistency and difficulty and then compiled into sections for the pre and post-intervention surveys. The questions were broken down into categories: personal finance (10), investing (11), real estate & mortgage (8), physician payment models (7), income & tax (7), and specialty salaries and career choice (3). The number of questions for each section was dependent on three variables: perception of need from literature, instructor feedback, and whether the content could be appropriately tested using multiple-choice questions. Through a literature search, we identified which financial topics were most deficient in medical students and resident physicians. We then collaborated with instructors and finalized the 46-question financial literacy examination. Financial wellness was examined through self-reported sentiments on items such as financial stress, biggest financial worry, budgeting practices, and retirement planning.

After the course, participants were asked about changes in their financial knowledge, confidence, attitude, and behavior. Lastly, participants were asked whether the MMM should be incorporated into medical education and to leave feedback.

Statistical methods and data analysis

Survey data were exported from Opinio into IBM Statistical Package for the Social Sciences software (IBM Corp. Released 2020. IBM SPSS Statistics for Windows, Version 27.0. Armonk, NY: IBM Corp). Financial literacy exam scores were used as a metric for general financial literacy among the cohort. Financial literacy scores were then compared to participant demographic factors using analysis of variance (ANOVA) and multivariate analysis of variance (MANOVA). Post-hoc testing using the Bonferroni method and homogeneity of variance with Levene’s test of equality of variances were applied, with statistical significance set at p ≤ 0.05. Paired T-tests assessed the significance of any changes to pre-intervention and post-intervention mean financial literacy scores.

Honorariums for study participants

A $10 CAD honorarium was given to participants for completion of each pre and post-intervention survey for a total of $20 CAD to encourage survey completion. To encourage live attendance and participation, a $50 raffle was held each week from the attendance list. $300 was disbursed in total for the six unique raffle winners throughout the course.

## Results

Demographics

Of the course participants, 64.86% (n=179/276) participated in the study and completed the pre-intervention survey. Ninety-three (93) of the 179 (51.96%) completed the post-intervention survey. Respondents’ age varied from 23 years to 39 years (SD = 3.29) (Table 2). Most respondents (78.8%, n = 141/179) came from urban communities (Table 3) defined as having a population greater than 10,000 [13]. Most respondents had no dependents (93.9%, n = 168/179). Most of the participants did not have a first-degree family member who is a physician (82.1%, n = 147/179). Most of the participants were first-year (M1) students (27.9%) and second-year (M2) students (26.3%) (Table 2).

**Table 2:** Parental education status of participants

Education	Maternal		Paternal	
	n	Percentage (%)	Frequency	Percentage (%)
Less than high school	4	2.2	3	1.7
High school	14	7.8	12	6.7
College or technical training	42	23.5	39	21.8
Bachelor	65	36.3	50	27.9
Master	22	12.3	25	14.0
Doctorate	4	2.2	16	8.9
Professional	34	19.0	39	21.8

**Table 3:** Participant demographics

		Pre-intervention			Post-intervention	
		n	Percentage (%)	Score	n	Percentage (%)
N (total)		179	100	-	93	100
Gender	Male	70	39.1	12.69 ± 6.90	36	38.7
	Female	107	59.8	11.24 ±5.89	57	61.3
	Non-binary	1	0.6	9.00	0	0
	Prefer not to say	1	0.6	9.00	0	0
Age	<25yo	58	32.4	10.40 ± 5.15	28	30.4
	26-30yo	95	53.1	12.28 ± 6.96	6	60.2
	>30yo	26	14.5	13.04 ± 5.79	9	9.7
Geography	Urban	141	78.8	10.08 ± 5.84	71	76.3
	Rural	38	21.2	12.24 ± 6.36	22	23.7
Marital status	Single	133	74.3	11.38 ± 6.33	69	74.2
	Married	20	11.2	13.80 ± 6.77	11	11.8
	Common law	25	14.0	12.44 ±5.73	13	14.0
	Separated	1	0	9.00	0	0
Year of study	M1	50	27.9	11.12 ± 6.13	23	24.7
	M2	47	26.3	11.09 ± 5.66	29	31.2
	M3	31	17.3	13.00 ±5.60	13	14.0
	M4	29	16.2	11.97 ± 8.07	18	19.4
	PGY1	8	4.5	13.38 ± 6.44	4	4.3
	PGY2	12	6.7	12.25 ±7.24	4	4.3
	PGY4	2	1.1	14.00 ±1.41	2	2.2
Education	Master	45	25.1	11.36 ± 6.22	21	22.6
	Doctorate	7	3.9	12.29 ± 2.56	4	4.3
	Professional	16	8.9	14.31 ± 6.98	9	9.7
Self-reported financial literacy	Poor	10	5.6	8.80 ± 6.23	2	2.2
	Not good	51	28.5	9.61 ± 5.33	13	14.0
	Average	71	39.7	11.39 ± 6.42	41	44.1
	Good	42	23.5	14.86 ± 5.37	32	34.4
	Excellent	5	2.8	19.60 ±7.02	5	5.4

Effect of demographics on financial literacy exam score

There were no notable differences in financial literacy exam scores per gender. Increased age had a very weak correlation with financial literacy exam scores that were not statistically significant (r = 0.152, p = 0.42). Whether the participant was a medical student or resident had no effect on financial literacy exam scores. Although urban participants had a greater baseline financial literacy exam score than rural participants, this was not significant (urban = 12.033 ± 1.062, rural = 9.685 ± 1.565, p = 0.216). Having a direct relative who is or was a physician had no impact on financial literacy exam scores (No first-degree physician = 12.619 ± 0.602, Yes first-degree physician = 11.994 ± 1.091).

Effect of financial literacy-related metrics on financial literacy exam score

In measuring baseline personal financial literacy, we explored subjective metrics such as self-reported financial literacy, financial stress, and retirement planning (Table 4). There was a positive correlation between self-reported financial literacy and scores on the exam (r = 0.366, p < 0.001). Participants who were planning for retirement (24%, n = 43/179) scored higher on the financial literacy exam than those who were not planning for retirement (mean difference of financial literacy exam scores = 5.919, p < 0.001).

**Table 4:** Effect of self-reported financial literacy, financial stress, and retirement plan on marginal mean financial literacy exam score

Self-reported financial literacy	n	%	Marginal mean score (95% CI)
Poor	10	5.6	9.31 (5.40-13.23)
Not good	51	28.5	9.49 (7.53-11.45)
Average	71	39.7	12.52 (10.73-14.32)
Good	42	23.5	15.36 (13.27-17.44)
Excellent	5	2.8	19.60 (14.30-24.90)
Financial stress	n	%	Marginal mean Score (95% CI)
Very high	4	2.2	12.25 (6.33-18.17)
High	12	12.8	9.42 (6.77-12.07)
Moderate	79	44.1	12.06 (10.26-13.86)
Low	66	36.9	14.67 (12.78-16.56)
Very low	7	3.9	11.17 (6.539-15.794)
Retirement plan	n	%	Marginal mean Score (95% CI)
Yes	43	24	15.06 (13.05-17.07)
No	136	76	10.69 (9.34-12.05)

There were significantly higher scores for those who self-reported “Excellent” compared to “Average”, “Not good”, and “Poor” (Excellent vs Average, p = 0.007; Excellent vs Not good, p = 0.001; Excellent vs Poor, p = 0.002). Reporting “Good” financial literacy also had a significant association with higher scores than lower ratings of “Average”, “Not good”, and “Poor” (Good vs Average, p = 0.004; Good vs Not good, p < 0.001; Good vs Poor, p = 0.003).

Participants who self-reported “Low” levels of financial stress scored the highest and showed significantly higher scores than the “High” and “Moderate” financial stress groups (Low stress vs High stress, mean difference = 5.254, p = 0.002; Low stress vs Moderate stress, Mean difference = 2.611, p = 0.05).

Effect of a financial education intervention on financial literacy

The MMM improved financial literacy scores by 10.10 (n = 93, p < 0.001), which is a mean increase of 22% on the financial literacy exam (pre-intervention score = 13.03 ± 6.11; post-intervention score = 23.13 ± 5.48). There was a strong paired correlation between pre and post-intervention scores, indicating that the MMM improved individual financial literacy (r = 0.614, p < 0.001).

Evaluation of Medical Mini-MBA

Beyond the value of improving financial literacy, subjective experiences were also recorded. Comments were overall positive, and numerous suggestions from the participants urged the content to be included in the medical education curriculum. Many comments also pointed out the paucity of financial knowledge within the medical training process.

Financial wellness: changes in knowledge, attitude, and behavior

We measured participants on whether they agreed that they experienced an improvement in financial knowledge, attitude, and behavior upon completion of the course. Participants agreed that their financial knowledge had improved (n = 93, median = 4 “Agree”, SD = 0.485). However, their attitude and behavior-changing were less impacted (n=93, median = 4 “Agree”, 4 “Agree”; SD = 0.910, 0.813; respectively).

Subjective experiences and suggestions for the Medical Mini-MBA

In total, 37 participants submitted text-based feedback (37.79%, n = 37/93). Most of the feedback suggested the course be implemented in the medical education curriculum (32%, n = 12/37). Others reported improved self-confidence and knowledge in personal finances, appreciating the expertise and experience of the instructors, praising the asynchronous offline viewing, and that it provided insight into a financial discussion in the medical profession.

## Discussion

Physicians may have difficulty making financial decisions throughout their career and may be poorly equipped to resolve personal financial problems [2,4,6-9,14]. This may be attributable to the many years of education and training to become a physician, a career that is academically arduous. Poor financial literacy may also be explained by the taboo of discussing finances as medical professionals, which was reported qualitatively in our course evaluation. From undergraduate education to becoming an attending physician and beyond, a structured method of learning and mastering financial literacy is lacking. Students and trainees recognize this issue along with administrators involved in the medical education and training process [8,10,11,15-16].

In this study, demographic factors, such as gender, geography of residence, and childhood financial stress, did not affect the financial literacy of students and residents. In Canada, there is evidence of a gender gap, albeit a complex one, in financial literacy [17-19]. With the changing of traditional gender roles, it is debated whether there are any significant differences among genders and financial literacy [18-19]. Conventional gender roles in domains such as employment, grocery shopping, purchasing decisions, marriage, real estate decisions, and other psychosocial factors have reduced the financial literacy gap in recent years [17-19]. Geography, particularly whether participants are situated rurally or in cities may not have an effect on financial literacy [20-21]. In Canada, rural medical students are typically older, come from families of lower socioeconomic status, have higher levels of debt, more often have part-time and summer employment, and have greater financial stress [22]. Despite this data, there was no difference in financial literacy scores between rural and urban cohorts of medical students. Financial stress during childhood and upbringing also had no impact on financial literacy scores. Having limited resources and being of low socioeconomic status was not correlated with poor financial literacy. Families with financial stress may be facing institutional and personal challenges, which are factors for financial stress but may not contribute to financial illiteracy [23].

The financial literacy exam was created by subject matter experts to accurately assess the knowledge of the participants on the instructed content material. The improvement was significant and demonstrates that a six-hour part-time course may improve the financial literacy of medical students. Participants were aware of their own deficits in financial literacy and financial decision-making. This is in line with previous studies, which show that confidence in financial literacy and decision-making is often a larger contributing factor than gender or other demographic factors [17].

The baseline score on the financial literacy exam before the intervention was 13.03/46 ± 6.30 for those who also completed the post-intervention exam. Although this is a low score of 28.33%, this is similar to other post-secondary rates of 27% for knowledge of interest rates, inflation, and risk diversification [24]. A post-intervention score of 23.13/46 yielded a 21.95% increase on the financial literacy exam (p < 0.001). This course was delivered as a voluntary extracurricular course that did not have protected time from regular extensive medical education and training. Protected time to invest in the financial wellness of medical students and residents may yield even more substantial improvements.

The MMM may be a method of improving financial literacy through gaining knowledge but attitude and behavior change needs further investigation. Targeting attitudes, such as motivation, and behavioral skills would be a logical step toward achieving financial wellness for medical students and residents [10].

In Canada, medical education is government-subsidized via public funding. However, burnout and financial challenges are rampant and well-being among physicians is currently at the forefront of investigation. We believe that better financial literacy will contribute to a reduction in financial stress and help improve physician wellbeing. This, in turn, will promote career longevity and career satisfaction and enhance the physician workforce. Ultimately, a robust physician workforce will contribute to better delivery of health care to our population. Thus, investment in a highly subsidized or no-cost financial literacy program with the support of administrators may be invaluable for wellbeing.

Additionally, by reducing the stress of learners, we postulate that anxiety imposed by financial stressors can be shifted toward medicine and delivering better care for patients [25]. Furthermore, with the “hidden curriculum” growing with topics such as billing and remuneration models, earlier exposure and unbiased instruction would benefit medical learners and enhance wellbeing [26-27]. Although integration into the medical curriculum may not be feasible for all institutions, financial education can augment the medical curriculum if offered to all learners. Furthermore, learners alike need to take the onus of seeking better financial literacy and education.

Limitations

A limitation of our study was the sample distribution among the medical student and resident population. Resident physicians only accounted for 3.35% of the cohort (n = 6/179). As the course was initially designed to improve financial wellness for medical students, additional courses or topics may be included in future iterations of the program to better serve resident physicians.

The current study did not assess retention of financial literacy or behavior change. Despite learning the material and being able to apply it to a multiple-choice exam, retention and real-life application are other scenarios that deserve further investigation. Investigators are planning an annual maintenance or refresher course to help with retention while elaborating on more advanced topics for students and resident physicians about to start their clinical practice.

Our instructors reported no conflicts of interest during their recruitment and again at the time of presentation to the study participants. Due to the nature of their expertise, many of our instructors are involved in finance-related professions, and they may have their own personal and professional biases. After an extensive review of their teaching material and recording each session, we do not believe there were any observable conflicts of interest.

## Conclusions

It is evident that medical students and resident physicians have deficits in financial literacy and are interested in improving the same. The MMM was successful in improving the financial literacy of participants and poses a solution to this problem. The MMM was well-received, and overwhelming feedback suggested it be incorporated into the medical education curriculum in some form. The benefits of a financial literacy intervention may include reducing stress throughout training, improved debt reduction strategies, and improved retirement planning. We believe early and sustained intervention can lead to long-lasting improvements in financial literacy and wellbeing. A financial literacy program may integrate into or augment the medical curriculum to assist today’s medical learners and equip them with better financial education.
